# Alzheimer disease neuropathology in a patient previously treated with aducanumab

**DOI:** 10.1007/s00401-022-02433-4

**Published:** 2022-05-17

**Authors:** Edward D. Plowey, Thierry Bussiere, Raj Rajagovindan, Jennifer Sebalusky, Stefan Hamann, Christian von Hehn, Carmen Castrillo-Viguera, Alfred Sandrock, Samantha Budd Haeberlein, Christopher H. van Dyck, Anita Huttner

**Affiliations:** 1 Research and Development, Biogen, 225 Binney Street, Cambridge, MA 02142 USA; 2 Department of Pathology, Yale University School of Medicine, New Haven, CT USA; 3 Department of Psychiatry, Yale University School of Medicine, New Haven, CT USA

**Keywords:** Alzheimer disease, Amyloid beta, Immunotherapy, Amyloid PET, Neuropathology, Case report

## Abstract

Amyloid beta (Aβ) plaque is a defining pathologic feature of Alzheimer disease (AD). Aducanumab, a monoclonal IgG1 that selectively binds aggregated species of Aβ, has been shown by amyloid positron emission tomography (Amyloid PET) to reduce Aβ plaques in patients with prodromal and mild AD. This is the first autopsy report of the AD neuropathology in a patient previously treated with aducanumab. The patient was an 84-year-old woman who was randomized to the placebo arm of the PRIME Phase 1b study (221AD103). The patient progressed to moderate dementia (MMSE = 14/30), beyond the targeted early AD treatment stage, before receiving aducanumab in the long-term extension (LTE). The patient then received 32 monthly doses of aducanumab, titrated up to 6 mg/kg, for a cumulative dose of 186 mg/kg. In the LTE, Amyloid PET scans demonstrated robust Aβ plaque reduction, from a composite standard uptake value ratio (SUVR) of 1.5 at screening to < 1.1 at 56 weeks post-aducanumab dosing. MRI examinations were negative for amyloid-related imaging abnormalities (ARIA). She passed away in hospice care 4 months after her last dose of aducanumab. The postmortem neuropathologic examination confirmed AD neuropathologic changes. Aβ and IBA1 immunohistochemistry assays demonstrated sparse residual Aβ plaque engaged by amoeboid reactive microglia. Phospho-Tau (pTau) immunohistochemistry demonstrated neocortical neurofibrillary degeneration (Braak stage V, NIA/AA Stage B3). However, the density of pTau neuropathology, including neuritic plaque pTau (NP-Tau), appeared lower in the PRIME LTE Patient compared to a reference cohort of untreated Braak stage V–VI, NIA/AA Stage B3 AD cases. Taken together, this case report is the first to provide Amyloid PET and neuropathologic evidence substantiating the impact of aducanumab to reduce Aβ plaque neuropathology in a patient with AD. Furthermore, this report underscores the critical importance of autopsy neuropathology studies to augment our understanding of aducanumab’s mechanism of action and impact on AD biomarkers.

**Supplementary Information:**

The online version contains supplementary material available at 10.1007/s00401-022-02433-4.

## Introduction

Alzheimer disease (AD) is the leading cause of chronic, progressive dementia. AD is characterized by two neuropathologic hallmarks: (1) extracellular plaques comprised of Amyloid beta (Aβ) fibrils and (2) intraneuronal aggregates of hyperphosphorylated Tau (pTau) that accumulate in neuritic plaques (NPs), neuropil threads (NTs) and neurofibrillary tangles (NFTs) [9, 15]. The pathogenesis of plaques and tangles and how they lead to the dementia syndrome remains to be fully elucidated. The amyloid cascade hypothesis, which is supported by genetic, pathologic and experimental data [11, 12, 30], posits that Aβ aggregates are the primary catalyst of neuroinflammation, Tau hyperphosphorylation, neurofibrillary degeneration and synapse impairment/loss leading to dementia. Amyloid positron emission tomography (Amyloid PET), which detects the defining Aβ plaque pathology of prodromal and mild AD, has been employed as a patient selection and pharmacodynamic response biomarker in recent clinical trials testing the amyloid cascade hypothesis [7, 33].

Aducanumab is a recombinant human monoclonal IgG1 (mAb) that was derived from peripheral B cells of healthy elderly subjects who lacked signs of cognitive impairment. Aducanumab binds to aggregated forms of Aβ, such as soluble oligomers, protofibrils and insoluble fibrils [1, 31] and induces dose-dependent reductions in Aβ plaque in Tg2576 mice through a microglia-mediated phagocytosis mechanism [1, 31]. Aducanumab has been granted accelerated approval by the US Food and Drug Administration (FDA) for the treatment of AD based on Aβ plaque reduction. Aβ plaque reduction has been demonstrated in patients with early symptomatic stages of AD (mild cognitive impairment due to AD and mild AD dementia) via reductions in Amyloid PET standard uptake value ratios (SUVR) in a Phase 1b (NCT01677572) [31] and two Phase 3 trials (NCT02477800 and NCT02484547) [6]. Postmortem histological evidence of Aβ plaque reduction by aducanumab in humans had not yet been reported.

Herein, we report the first neuropathologic analysis of brain tissue samples from a patient previously enrolled in the aducanumab Phase 1b PRIME study. The patient was randomized to the placebo arm during the first 12 months of the study and progressed to moderate dementia during that period, beyond the targeted early AD treatment stage. The patient subsequently enrolled in the PRIME Long Term Extension (LTE), and then was administered aducanumab. Amyloid PET scans demonstrated Aβ plaque reduction upon aducanumab treatment, during the first year of the LTE. During the LTE, the patient progressed from her advanced state of moderate dementia, when aducanumab treatment was started, to end-stage dementia. The patient discontinued aducanumab in hospice care and, 4 months after her final dose of aducanumab, the patient passed away and her brain was donated to the Yale Alzheimer Disease Research Center for neuropathologic examination.

Consensus neuropathologic examination according to National Institute on Aging/Alzheimer Association (NIA/AA) guidelines [18] confirmed the presence of AD neuropathologic changes and absence of significant comorbid neuropathology. Aβ immunohistochemistry (IHC) assays demonstrated sparse residual Aβ plaque with morphologic features consistent with active plaque removal by microglia. Immunohistochemistry for phosphorylated tau protein (pTau) demonstrated neocortical neurofibrillary degeneration (Braak stage V, NIA/AA stage B3). However, the density of pTau neuropathology, including neuritic plaque Tau (NP-Tau), appeared lower than a control cohort of tissue samples from untreated Braak stage V–VI AD patients. These histopathological findings in a single AD patient treated with aducanumab provide the first neuropathologic evidence of Aβ plaque reduction by aducanumab. Furthermore, they underscore the critical importance of autopsy neuropathology studies to better understand aducanumab’s mechanism of action (MOA) in the human brain and impact on AD biomarkers.

## Materials and methods

### Gross examination, histology and immunohistochemistry (IHC)

The neuropathologic assessment, which was conducted according to the NIA/AA consensus guidelines for the neuropathologic assessment of AD [18], was performed by a Board-certified neuropathologist (AH) at Yale University. The brain and spinal cord were fixed in 10% neutral buffered formalin for 3 weeks. Brain tissue samples were processed and embedded into paraffin blocks, sectioned on a microtome at 5 μm thickness, mounted on to plus-charged slides and stained with hematoxylin and eosin.

Immunoperoxidase stains with the following primary antibodies were performed at Yale University Department of Pathology: Aβ (clone 6F/3D, epitope 8–17; Dako, M0872; dilution 1:100; formic acid pre-treatment for 6 min); phospho-Tau^Ser202,Thr205^ (AT8, Invitrogen, MN1020; dilution 1:2000); α-synuclein (Millipore, AB5038; dilution 1:2000; CC1 antigen retrieval for 24 min). Staining was performed on a Ventana Benchmark Ultra autostainer.

Additional IHC assays were performed at Biogen in the Translational Neuropathology Laboratory on a Ventana DISCOVERY Ultra automated stainer. The following primary antibodies were used: total Aβ (clone 6E10, epitope 1–16; BioLegend, 803002; 1.0 µg/mL; 88% formic acid antigen retrieval for 3 min); ^Ch^aducanumab (clone 12F6A, epitope 3–7; Biogen, PQ14409-126; 0.28 µg/mL; CC1 antigen retrieval for 16 min); Aβ_1–42_ (clone 12F4; BioLegend 805501; 1.0 µg/mL; 88% formic acid antigen retrieval for 3 min); Aβ_1–4o_ (clone 11A50-B10; BioLegend 805401; 62.5 ng/mL; 88% formic acid antigen retrieval for 3 min); phospho-Tau^Ser202,Thr205^ (^Ch^40E8; Biogen, R104W; 0.25 µg/mL; CC1 antigen retrieval for 64 min) [24]; phospho-Tau^Ser202,Thr205^ (AT8; Invitrogen, MN1020; 0.125 µg/mL; CC1 antigen retrieval for 64 min); phospho-Tau^Thr181^ (AT270; Invitrogen, MN1050; 0.125 µg/mL; CC1 antigen retrieval for 64 min); phospho-TDP-43^Ser409/410^ (Clone 11-9; Cosmo Bio USA, CAC-TIP-PTD-M01; 1:20,000; CC1 antigen retrieval for 40 min); Human IgG (H + L) (Jackson ImmunoResearch Laboratories, 309-005-003; 0.125 µg/ml; Protease 1 antigen retrieval for 8 min). The chimeric versions of the human antibodies 12F6A and 40E8, which feature murine Fc domains, were used to eliminate background that would occur from the use of a secondary anti-human IgG in human brain sections. The anti-HuIgG IHC assay, which was titrated to minimize endogenous HuIgG reactivity in all untreated AD control samples, was attempted to detect aducanumab target engagement. A dual Aβ(6E10)/IBA1 IHC assay (Aβ: clone 6E10; BioLegend, 803002; 0.25 µg/mL; 88% formic acid antigen retrieval for 3 min/IBA1: Wako, 019-19741; 0.125 µg/mL; CC1 antigen retrieval for 64 min) was employed to analyze microglial recruitment and reactivity to Aβ plaques. A dual Aβ/^Ch^40E8 IHC assay (Aβ: clone H31L21; Invitrogen, 700254; 0.0625 µg/mL; CC1 antigen retrieval for 32 min; ^Ch^40E8: Biogen, R104W; 0.125 µg/mL; CC1 antigen retrieval for an additional minutes) was employed to segment and quantify neuritic pTau within Aβ plaques (NP-Tau). The Perls iron method was performed to evaluate for remote hemorrhages/hemosiderin. All IHC and histochemical assay runs included sections from all control cases simultaneously to preclude batch effects.

Neuropathologic markers were compared between the PRIME LTE Patient and a reference AD cohort that was comprised of 2 brain tissue donors with HIGH AD neuropathologic changes from the Yale Alzheimer Disease Research Center (ADRC) research cohort and 7 brain tissue samples from donors with HIGH AD neuropathologic changes obtained from Netherlands Brain Bank (NBB), Netherlands Institute for Neuroscience Amsterdam (open access www.brainbank.nl). All materials have been collected from donors for or from whom a written informed consent for a brain autopsy and the use of the material and clinical information for research purposes has been obtained by the NBB. Characteristics of untreated AD control cases are presented in Table S1.

### Whole slide image (WSI) analysis

Slides were digitized on a Pannoramic P250 whole slide scanner using a 20 × objective. WSIs were analyzed with custom-designed segmentation and quantitation algorithms in Visiopharm Image Analysis Software (version 2019.12.0.6842). Cortical regions of interest (ROIs) were annotated manually. Aβ and pTau immunoreactivity densities (area immunoreactivity/area ROI, expressed as %) were quantified with threshold-based algorithms using median filters and respective color deconvolution filters. Shape-sensitive post-processing steps were employed to exclude vascular Aβ from Aβ plaque segmentations. Heatmaps of Aβ plaque and pTau neuropathology densities were created using Visiopharm software visualizing accumulated object area. Microglia recruitment to Aβ plaques was quantified by first segmenting 6E10 and IBA1 immunoreactivities and then measuring the percentage of IBA1 area within 5 µm radii of Aβ plaque. NP-Tau was quantified as the % Area of pTau immunoreactivity within segmented Aβ plaque area. Data were graphed using GraphPad Prism v. 7.02 software.

## Results

### Case presentation

**Fig. 1:**
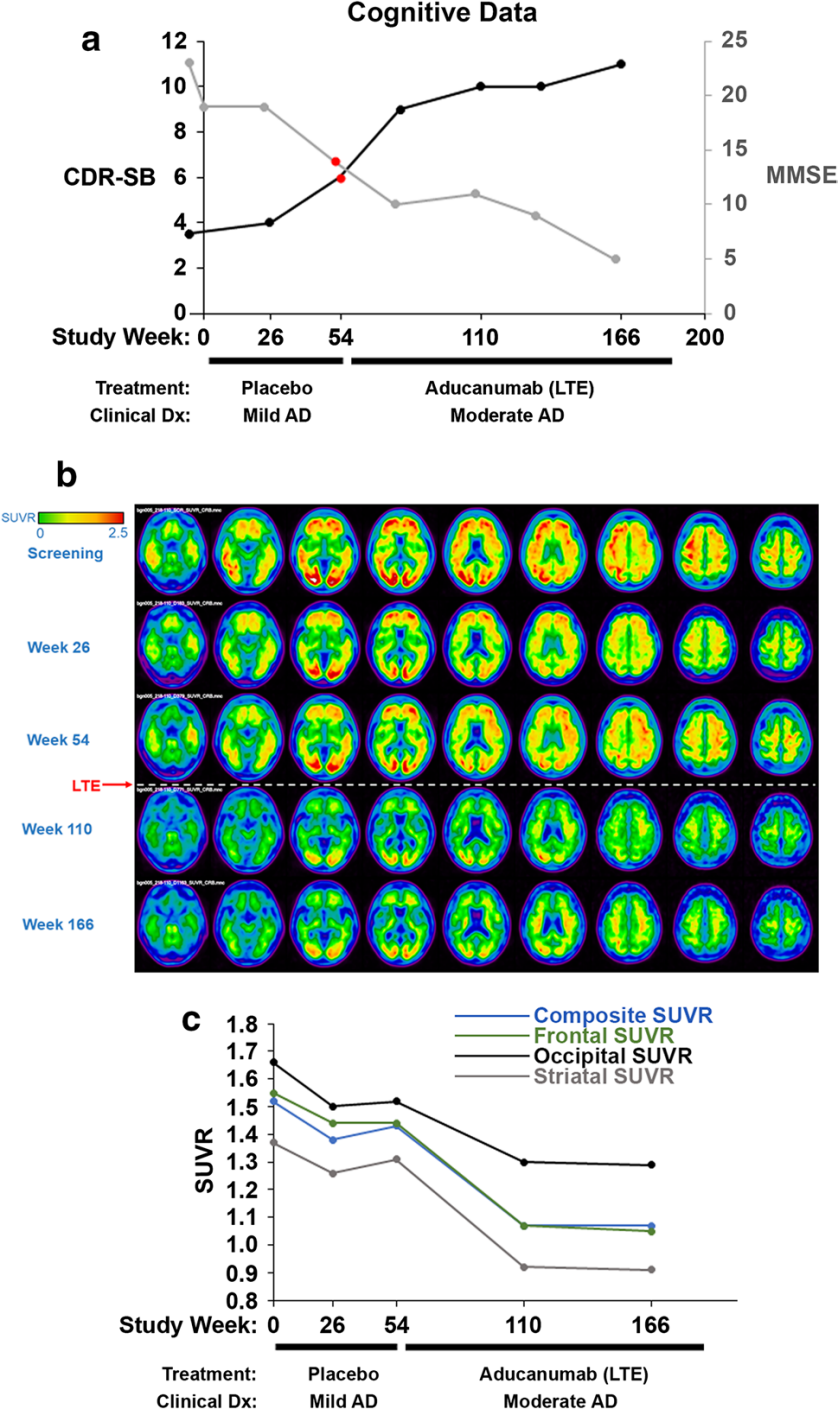
Cognitive progression and Amyloid PET biomarker data during the Phase 1b PRIME Study and LTE. **a** CDR-SB (y-axis, left) and MMSE (y-axis, right) progression from initial patient screening through the Phase 1b (placebo) and the LTE (aducanumab). Scores at screening are shown to the left of the left y-axis. The cognitive data points highlighted in red are those measurements that immediately preceded initiation of aducanumab administration and are consistent with moderate dementia prior to enrollment in the LTE. **b** Axial slice 18F-florbetapir PET images at baseline/screening (top row), Weeks 26 and 54 (rows 2 and 3) in the Placebo arm, and at Weeks 110 and 166 (rows 4 and 5) of the LTE demonstrate reduction in standardized uptake value ratio (SUVR), indicative of Aβ plaque reduction, following administration of aducanumab (red arrow). **c** Composite and regional SUVRs, graphically presented, demonstrate Amyloid plaque reduction in frontal cortex, occipital cortex and striatum. Note that the occipital cortex demonstrated the highest residual Amyloid plaque among these regions

The patient was an 84-year-old woman who was diagnosed with mild AD in 2010 after seeking evaluation for complaints of recent memory loss, word-finding difficulty and trouble balancing her checkbook. Her earliest symptoms had appeared approximately two years earlier and progressed gradually. Her *APOE* genotype was *E3*/*E3*. Her past medical history included coronary artery disease (history of coronary artery stent), hypertension, hyperlipidemia, and depression. Her medications included rivastigmine transdermal patch and memantine. She screened for the PRIME Phase 1b study in 2013. Screening cognitive data included an MMSE score of 23, corresponding to mild cognitive impairment (Fig. 1a). An Amyloid PET scan demonstrated Aβ plaque throughout the cerebral cortex and striatum (Fig. 1b, c). She was randomized to the placebo arm of the PRIME Phase 1b Trial during which she received 14 intravenous infusions of placebo. The patient demonstrated progression of her cognitive impairment during placebo treatment, with scores of 6 on the CDR-SB and 14 on the MMSE at week 54, consistent with moderate dementia (Fig. 1a).

The patient then enrolled in the LTE, during which she received 2 monthly doses of aducanumab 3 mg/kg IV followed by 30 monthly doses of 6 mg/kg IV. During this time, all per-protocol MRIs were negative for amyloid-related imaging abnormalities (ARIA). Amyloid PET studies at 110 and 166 weeks (occurring 56 and 112 weeks following the start of aducanumab dosing, respectively) demonstrated SUVR reductions in the frontal, temporal and parietal cortices and striatum compared to baseline and Weeks 26 and 54 scans during the placebo treatment (Fig. 1b, c). SUVR signal declined in the occipital cortex during aducanumab dosing but remained elevated compared to the other cortical regions. The patient progressed from moderate dementia to end-stage dementia (MMSE of 5/30) and entered skilled nursing care. The patient passed away 4 months after her last dose of aducanumab. A brain donation autopsy was performed following a 17-h postmortem interval.

### Neuropathologic examination

Gross brain examination revealed a total brain weight of 1000 g. The leptomeninges showed no hemorrhage or hemosiderin. There was bilaterally symmetrical cortical atrophy involving the frontal, temporal and parietal lobes. Coronal sections demonstrated bilaterally symmetrical hippocampal atrophy. The substantia nigra was normally pigmented. There were no recent or remote infarcts or parenchymal hemorrhages.

According to NIA/AA consensus guidelines [18], tissue sections confirmed the presence of Alzheimer disease neuropathologic changes: Aβ plaques were observed in neocortex and hippocampus but not in the striatum (Thal Phase 2); NFTs were observed in sections of association neocortex (Braak stage V); and sparse neocortical NPs were observed on Tau immunostained sections (CERAD score 1). The composite NIA/AA ABC score was A1, B3, C1, consistent with “Low AD Neuropathologic Changes”. Granulovacuolar degeneration was observed only in the CA1, CA2 and subicular subfields (Stage 1). There was no significant glial tauopathy, no ballooned neurons or other neuropathologic signs of non-Alzheimer tauopathy. The neuropathologic examination was also negative for the following: Lewy bodies, TDP-43 proteinopathy (pTDP-43 IHC performed on parahippocampal gyrus, hippocampus, frontal and temporal neocortex), hippocampal sclerosis and microinfarcts.

**Fig. 2:**
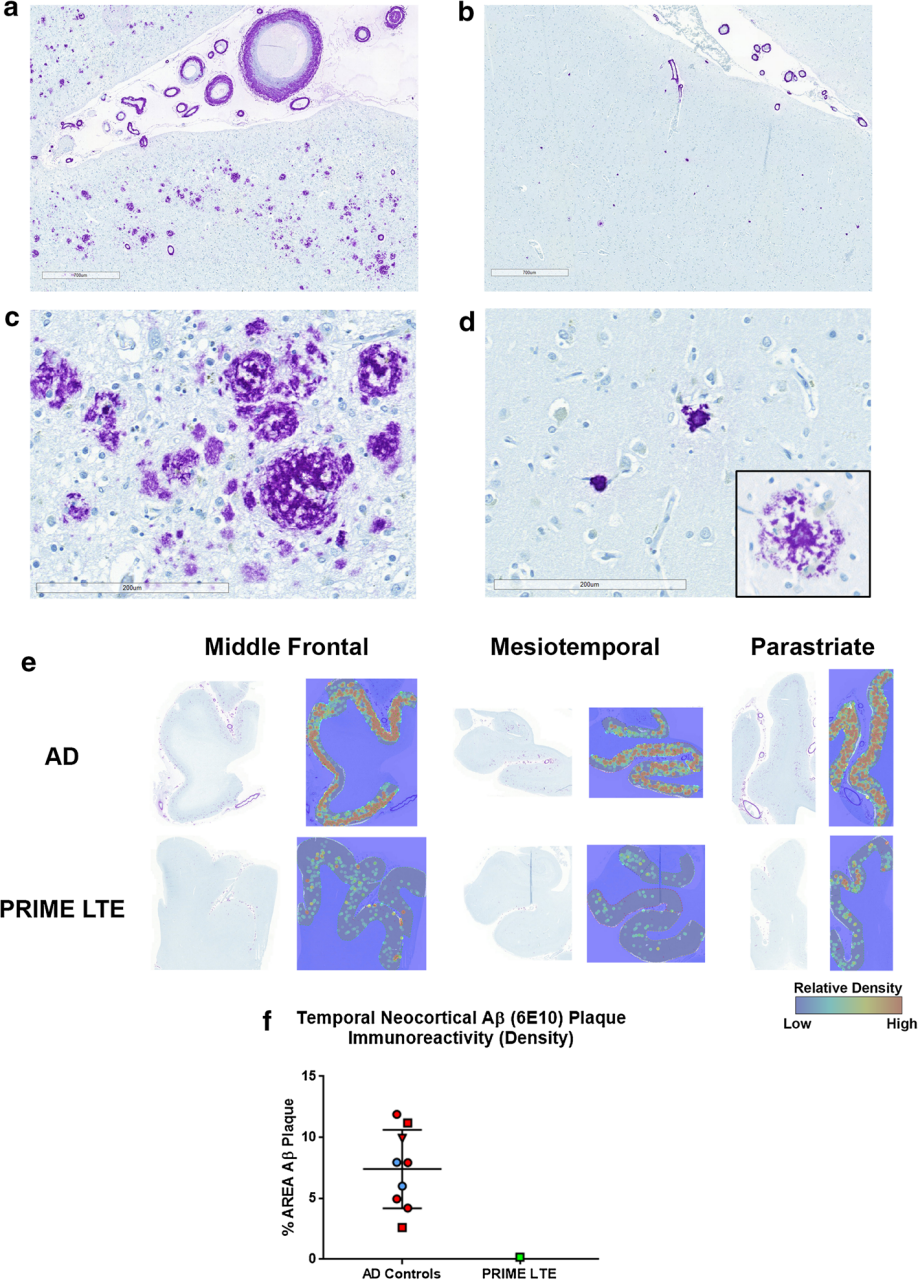
Aβ immunohistochemical stains demonstrate sparse residual Aβ plaques comprised predominantly of dense cores following aducanumab treatment. **a**–**d** Low- and high-power magnification images of frontal neocortex from an untreated HIGH AD neuropathology case of the Yale ADRC research cohort **a**, **c** demonstrating frequent cortical Aβ plaques (6E10, purple) and amyloid angiopathy. Images from the PRIME LTE subject **b**, **d** demonstrating sparse cortical Aβ plaque and amyloid angiopathy. Aβ plaques were predominantly comprised of dense cores surrounded by reactive microglia. Dense cores surrounded by moth-eaten peripheral halos of non-compact Aβ with reactive microglia were most prevalent in the occipital neocortex (inset **d**). Low-power image original magnification ×33, scale bars 700 µm; high-power image original magnification ×200, scale bars 200 µm. **e** Heat maps generated from 6E10-immunostained sections of middle frontal (left panels), mesiotemporal (middle panels) and parastriate cortices (right panels) demonstrate lower Aβ plaque burden throughout sections in the PRIME LTE Subject (lower row) compared to the HIGH AD neuropathology control (upper row). Note that the highest density of residual Aβ plaque was found in the parastriate neocortex consistent with the Amyloid PET SUVR data in Fig. 1b, c. **f** A graphical comparison shows a very low density of temporal neocortical Aβ plaque in the PRIME LTE Subject compared to a higher range of temporal neocortical Aβ plaque burden in a cohort of 9 HIGH AD case controls. Blue datapoints denote samples from Yale; red datapoints indicate samples from NBB. Squares denote *APOE4* non-carriers; circles denote *APOE4* allele carriers; the triangle denotes a sample with unknown *APOE* genotype

Aβ immunohistochemical stains revealed frequent cortical Aβ plaques in a cohort of control sections from untreated Braak stage V-VI AD cases (Fig. 2a, c). Cortical sections from the PRIME LTE Patient (Fig. 2b, d), in contrast, showed sparse residual Aβ plaque morphologically comprised predominantly of dense cores that lacked rims of non-compact Aβ (Fig. 2d). In rare plaques where peripheral halos of non-compact Aβ persisted, particularly in sections of the parastriate cortex, they demonstrated a moth-eaten appearance with conspicuous reactive microglia (Fig. 2d, inset). Whole slide image heatmaps of cortical Aβ plaques generated in Visiopharm demonstrated the sparsity of Aβ plaque throughout sections of frontal, mesiotemporal and occipital cortex in the PRIME LTE Patient compared to sections from HIGH AD case controls (Fig. 2e). The highest density of residual Aβ plaque, in concordance with the Amyloid PET SUVR data, was present in sections of the occipital cortex (Fig. 2e, right panels). Sections of the basal ganglia and midbrain were devoid of Aβ plaques. We compared temporal neocortical Aβ plaque density in the PRIME LTE Patient with a cohort of HIGH AD case controls (Fig. 2f), finding that Aβ plaque burden was markedly lower in the PRIME LTE Patient (% area of 0.17%). Taken together, these ex vivo neuropathologic findings confirm the Aβ plaque reduction demonstrated by Amyloid PET in this patient.

Cerebral amyloid angiopathy (CAA), non-capillary type, Vonsattel Grade 2 (full thickness mural Aβ deposition with some medial smooth muscle cell loss but no evidence of mural cracking or necrosis [10]), was present in the leptomeninges and cortex (Fig. 2b), even in areas fully devoid of Aβ plaques (Fig. S1a). A dual 6E10/IBA1 assay demonstrated only rare leptomeningeal macrophages in the vicinity of amyloid-laden cortical arterioles and no evidence of Aβ-related angiitis (Fig. S1b). There was no evidence of cortical or subcortical hemorrhages, microinfarcts or parenchymal or leptomeningeal siderosis in the PRIME LTE Patient. Perls iron stains showed no increase in cortical and leptomeningeal iron in the PRIME LTE Patient compared to a cohort of HIGH AD case controls (Fig. S1c, d). ^Ch^aducanumab immunoreactivity appeared lower than 6E10 immunoreactivity in CAA lesions but appeared comparable to 6E10 immunoreactivity in residual plaques with the employed protocol (Fig. S1e, f).

Our HuIgG IHC assay did not reveal clear evidence of aducanumab bound to residual plaques or CAA (Fig. S2). The assay, which was titrated to minimize endogenous parenchymal HuIgG immunoreactivity in all 9 untreated AD control cases, may not have been sensitive enough to detect an expected low level of aducanumab target engagement considering the 4-month interval since the Patient’s final aducanumab infusion.

We employed Aβ_1–42_, Aβ_1–4o_ and 6E10 immunohistochemistry protocols in near adjacent sections of frontal cortex from the PRIME LTE patient to detect potential differences in N-terminal and C-terminal Aβ epitope availability (Fig. S3). We found similar labeling of cortical Aβ plaque burden with 6E10 and Aβ_1–42_ IHC protocols (6E10: 0.09%; Aβ_1–42_: 0.04%). Aβ_1–4o_ labeling was absent in cortical Aβ plaques but was comparable to 6E10 labeling in cortical and leptomeningeal CAA. We saw very little Aβ_1–42_ immunoreactivity in cortical and leptomeningeal CAA, providing no evidence in this patient of vascular Aβ_1–42_ deposition secondary to perivascular drainage of Aβ removed from the parenchyma upon treatment with aducanumab.

**Fig. 3:**
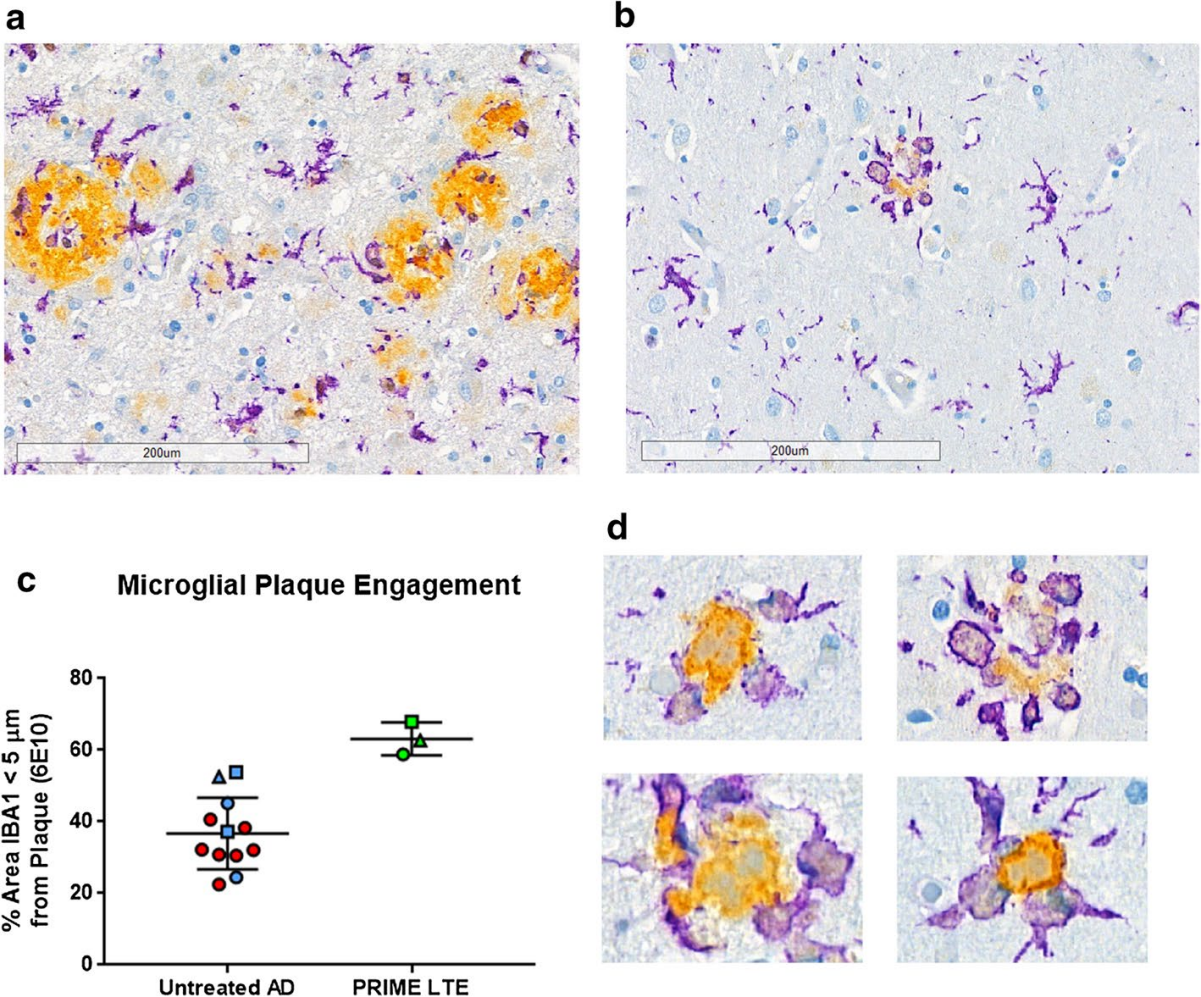
Microglia surrounding residual dense core Aβ plaques demonstrate amoeboid reactive morphology. **a**, **b** Low-power images (original magnification ×200, scale bars 200 µm) of sections from a HIGH AD case control (**a**) and the PRIME LTE Subject (**b**) reacted with a duplex 6E10/IBA1 IHC staining protocol. **c** Quantitation of IBA1 immunoreactive processes within 5 microns of segmented Aβ plaque demonstrates increased plaque engagement by microglia in the PRIME LTE Patient. Blue symbols denote cases from Yale; red symbols indicate cases from NBB. Circles indicate data from temporal lobe sections; squares indicate data from frontal lobe sections; triangles indicate data from occipital lobe sections. **d** Microglia (IBA1, purple chromogen) surrounding residual dense core Aβ plaques (yellow chromogen) show reactive amoeboid morphology

We employed a dual 6E10/IBA1 IHC assay to examine microglial reactivity to residual Aβ plaques (Fig. 3). Compared to sections from a cohort of HIGH AD case controls (Fig. 3a), microglia with highly reactive amoeboid morphology showed close association with residual dense cores and moth-eaten plaques in the PRIME LTE Patient (Fig. 3b). A WSI analysis algorithm that we designed to segment and quantify microglial IBA1 immunoreactivity within 5-µm radii of Aβ plaque edges demonstrated higher microglial plaque engagement in the PRIME LTE Patient compared to a series of untreated HIGH AD patients (Fig. 3c). High-power views from the PRIME LTE Patient samples demonstrated Aβ plaque surrounded by microglial processes and amoeboid reactive microglia (Fig. 3d).

**Fig. 4:**
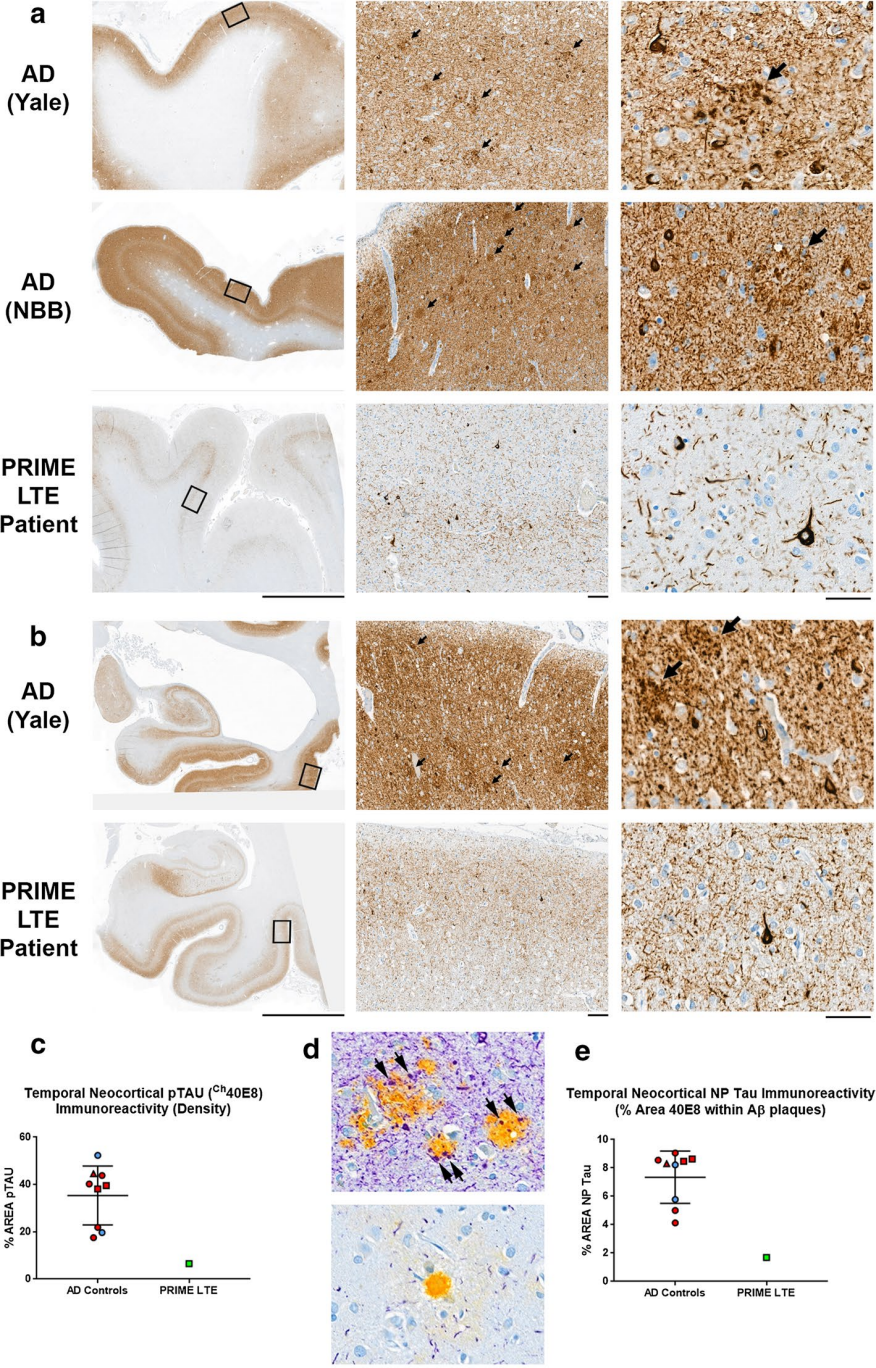
Phosphorylated Tau (pTau; 40E8) immunohistochemistry demonstrates sparse neocortical NPs in the PRIME LTE subject. **a** Sections of frontal neocortex from a HIGH AD neuropathology case control from the Yale ADRC research cohort (top row), a HIGH AD neuropathology case control from the Netherlands Brain Bank (NBB, middle row) and the PRIME LTE Subject (bottom row). Left column: low-power images (original magnification ×2.5, scale bar 5 mm) show dense pTau immunohistochemical reactivity in the HIGH AD sections from Yale and NBB compared to the PRIME LTE Subject. Middle column: medium power images (original magnification ×33, scale bar 100 µm) of the regions identified by boxes in the left column demonstrating frequent NPs (arrows) in HIGH AD case controls but no NPs in the PRIME LTE Subject section. Right column: high-power images (original magnification ×140, scale bar 50 µm) demonstrating NPs (arrows), frequent NFTs and dense NTs in HIGH AD case controls from Yale and NBB. The section from the PRIME LTE Patient shows comparatively fewer NFTs and NTs. **b** Section of mesiotemporal lobe including hippocampus, parahippocampal gyrus and occipitotemporal gyrus from a HIGH AD case control from Yale (top row) and the PRIME LTE Subject (bottom row). Left column: low-power images (original magnification ×2.5, scale bar 5 mm) show dense pTau immunohistochemical reactivity in the occipitotemporal neocortex in the HIGH AD case control from Yale compared to the PRIME LTE Subject. Reactivity in the parahippocampal gyrus is more comparable in these 2 cases. Middle column: medium power images (original magnification ×33, scale bar 100 µm) of the occipitotemporal neocortical regions identified by boxes in left column demonstrating frequent NPs (arrows) in HIGH AD sections from Yale but no NPs in the PRIME LTE Subject section. Right column: high-power images (original magnification ×140, scale bar 50 µm) demonstrating NPs (arrows), frequent NFTs and dense NTs in HIGH AD sections from Yale. The section from the PRIME LTE Patient shows comparatively fewer NFTs and NTs. **c** A graphical comparison shows a low density of temporal neocortical pTau neuropathology in the PRIME LTE Subject compared to a higher range of temporal neocortical pTau neuropathology in a cohort of 9 HIGH AD case controls. Blue datapoints denote samples from Yale; red datapoints indicate samples from NBB. Squares denote *APOE4* non-carriers; circles denote *APOE4* allele carriers; the triangle denotes a sample with unknown *APOE* genotype. **d** Representative images of pTau immunoreactivity in NPs. Top panel: NPs in HIGH AD patients demonstrate the pTau-immunoreactive dystrophic neurites (black arrows). Lower panel: residual dense core amyloid plaques, in contrast, often do not show pTau-immunoreactive dystrophic neurites in the PRIME LTE Subject. **e** A graphical comparison shows a low density of temporal neocortical NP-Tau neuropathology in the PRIME LTE Subject compared to a higher range of temporal neocortical NP-Tau neuropathology in a cohort of 9 HIGH AD case controls. Blue datapoints denote samples from Yale; red datapoints indicate samples from NBB. Squares denote *APOE4* non-carriers; circles denote *APOE4* allele carriers; the triangle denotes a sample with unknown *APOE* genotype

Phosphorylated Tau^Ser202,Thr205^ (pTau; ^Ch^40E8 antibody) IHC assays revealed neurofibrillary degeneration in sections of association neocortices from the PRIME LTE Patient (Fig. 4), indicative of Braak stage V/VI (NIA/AA stage B3) neurofibrillary degeneration. However, compared to sections from a cohort of HIGH AD case controls (Fig. 4a, top and middle rows; Fig. 4b, top row), sections of frontal and occipitotemporal neocortex from the PRIME LTE Patient were remarkable for lower pTau neuropathology (Fig. 4a, b, bottom rows). In the PRIME LTE Patient, pTau immunoreactivity appeared higher in the parahippocampal gyrus and focally in the CA1 sector compared to frontal and occipitotemporal neocortices (Fig. 4a, b). A WSI analysis algorithm designed to segment and quantify pTau immunoreactivity demonstrated lower temporal neocortical pTau density in the PRIME LTE Patient compared to the range of the cohort of HIGH AD case controls (Fig. 4c). Similar findings were evident with pTau IHC assays using commercially available pTau^Ser202,Thr205^ (AT8) and pTau^Thr181^ (AT270) antibodies (Fig. S4). We employed a dual Aβ (H31L21)/pTau^Ser202,Thr205^ (^Ch^40E8) IHC assay to demonstrate pTau-immunoreactive dystrophic neurites associated with Aβ plaques in HIGH AD case controls (neuritic plaque Tau or NP-Tau; Fig. 4d, top panel). NP-Tau was not apparent around most residual plaques in neocortical sections from the PRIME LTE Patient (Fig. 4d, bottom panel). A WSI analysis algorithm designed to segment and quantify NP-Tau immunoreactivity demonstrated lower temporal neocortical NP-Tau density in the PRIME LTE Patient compared to the range of the cohort of HIGH AD case controls (Fig. 4e).

## Discussion

Herein, we report the first human clinical-neuropathologic correlation demonstrating that aducanumab reduced Aβ plaque in an AD patient enrolled in the PRIME study and LTE. At screening and during the placebo-controlled period of PRIME, during which the Patient was randomized to placebo, Amyloid PET SUVRs ranged from 1.5 to 1.7 in cortical regions and registered 1.4 in the striatum, consistent with at least a Thal Phase 3 of Aβ plaque deposition [32]. During the LTE, the subject received 2 monthly doses of 3 mg/kg and then 30 monthly doses of 6 mg/kg of aducanumab, causing SUVRs to drop to < 1.1 in these regions in the first 54 weeks of the LTE. Aβ IHC assays employing antibodies 6E10 (which notably is not blocked from detecting Aβ plaque by aducanumab [31]), 6F/3D, ^Ch^12F6A and Aβ_1–42_ were used to detect residual plaques in tissue sections. All assays demonstrated a paucity of Aβ plaque in sections of the frontal and temporal cortices and absence of Aβ plaque in sections of the striatum. In the occipital cortex where neuropathologic examination also confirmed the greatest residual Aβ plaque burden in this patient, Amyloid PET SUVR was reduced compared to baseline but remained elevated compared to frontal and temporal cortices. Compared to a reference cohort of untreated control Braak stage V–VI AD cases from the Yale ADRC and NBB, immunohistochemical Aβ plaque labeling in the PRIME LTE Patient was markedly lower. These neuropathologic data support Amyloid PET as a pharmacodynamic biomarker demonstrating Aβ plaque reduction by aducanumab in a Patient with AD.

The morphology of the sparse residual Aβ plaque in the PRIME LTE Patient—dense cores lacking peripheral halos of non-compact Aβ and moth-eaten plaques with conspicuous reactive microglia—was distinctive compared to the reference HIGH AD cases used in this study. These residual Aβ plaque morphologies are similar to those previously reported for active immunization with AN-1792 in patients with mild-to-moderate AD [5, 14, 21, 22]. AN-1792 was an investigational full-length Aβ_1–42_ peptide administered with QS-21 adjuvant that was halted in Phase 2a of clinical development due to meningoencephalitis [25]. In long-term follow-up spanning up to 14 years, donated brains from 14/16 neuropathologically confirmed AD patients administered AN-1792 demonstrated evidence of long-lasting plaque removal. The reported AN-1792 immunized patients predominantly showed patchy Aβ plaque removal in most cases. Only 5/14 AN-1792 immunized patients showed “very extensive plaque removal” and lacked patchy regions with frequent residual plaques [22]. In the PRIME LTE Patient reported herein, heatmaps of residual Aβ plaque are consistent with relatively confluent plaque reductions. There was no neuropathologic evidence of vasculitis or meningoencephalitis in the PRIME LTE Patient.

Another notable difference between the PRIME LTE Patient and the findings reported in AN-1792 immunized patients is the conspicuous reactive amoeboid microglial morphology we observed with aducanumab treatment. Microglia are major effectors of passive Aβ immunotherapy via FcR-mediated phagocytosis of Aβ-antibody complexes [2]. ^Ch^aducanumab induces amoeboid microglia to engage and phagocytose Aβ plaques in Tg2576 mice, whereas aglycosylated ^Ch^aducanumab with attenuated effector function shows reduced potency to induce these changes, further implicating Fc receptor-mediated phagocytosis as a significant clearance mechanism [31]. Consistent with a microglial effector mechanism for aducanumab in humans, we observed microglia with highly reactive, amoeboid morphology surrounding residual plaque cores and infiltrating moth-eaten plaques in the PRIME LTE Patient. Microglia showed increased Aβ plaque engagement in the PRIME LTE Patient compared to the reference cohort of HIGH AD cases. Microglial Aβ phagocytosis was also demonstrated in patients immunized with AN-1792, yet microglia in AN-1792 immunized patients appear more ramified than the amoeboid microglia we observed in the PRIME LTE Patient [20, 22]. The morphology we observed is consistent with Aβ plaque engagement and removal by microglia.

Published neuropathologic studies from patients immunized with Aβ antibodies are unfortunately sparse and provide scant substrate for comparison to our findings with aducanumab in terms of amyloid removal. Three autopsies from AD patients treated with bapineuzumab, a humanized monoclonal antibody (3D6) that binds to the N-terminus of fibrillar, oligomeric and monomeric Aβ, showed similar levels of Aβ plaques, CAA and neurofibrillary degeneration similar to non-immunized controls [27]. Two of these patients were *APOE ε4* carriers that stopped dosing after 4 or fewer bapineuzumab doses (0.5–2.0 mg/kg) due to ARIA. One *APOE ε2/ε3* patient received 20 doses of 1.0 mg/kg of bapineuzumab over 260 weeks. Similarly, a dearth of diffuse Aβ plaques in the cortex and striatum but no reduction in cortical NPs was described in an abstract presenting one patient who received 4 doses of bapineuzumab (1 mg/kg) [17]. However, SUVR reductions in Phase 3 bapineuzumab trials of about 0.1 [16] are substantially lower compared to the reduction of 0.3 in our PRIME LTE Patient and compared to the mean SUVR reductions observed in the PRIME study [31]. A report of a 79-year-old male who received 9 months of treatment with solanezumab showed no apparent differences between total plaque and NFT scores compared to non-immunized AD patients [28]. Unlike aducanumab, solanezumab binds to soluble monomeric—not fibrillar deposited—Aβ and has been reported to have no significant impact on 18F-florbetapir PET SUVR in patients with baseline and Week 80 follow-up studies [8].

Neuropathologic examination of the PRIME LTE Patient also disclosed mild CAA, a near universal neuropathologic finding in patients with AD. However, considering the paucity of parenchymal Aβ plaques, the amyloid-laden arterioles were unusually conspicuous. Augmented vascular Aβ deposition has also been hypothesized to occur with Aβ vaccination in humans [4], and if generalizable to passive immunization in humans could explain the observation of CAA in cortical regions nearly devoid of plaques in the PRIME LTE Patient. However, the paucity of Aβ_1–42_ immunoreactivity in CAA lesions of the PRIME LTE Patient provide no clear support for vascular Aβ_1–42_ deposition secondary to perivascular drainage of Aβ removed from the parenchyma in this case. CAA is thought to be an important pathology that may be related to ARIA, microinfarcts and Aβ-related vasculitis, all of which did not occur in this PRIME LTE Patient. Using an IHC assay with ^Ch^aducanumab as the primary antibody, we observed less immunoreactivity with vascular Aβ in the PRIME LTE Patient compared to immunoreactivity with the residual Aβ plaques, similar to findings in Tg2576 mice [31]. The relevance of such findings remains to be clarified.

Our observation of low neocortical pTau neuropathology and low neocortical NP-Tau in the PRIME LTE Patient compared to the reference cohort of HIGH AD cases notably parallels the reduction in the progression of Tau PET, CSF pTau and plasma pTau^181^ biomarkers in Phase 3 ENGAGE and EMERGE studies [6]. Aβ plaques accelerate pTau neuropathology in preclinical models [3, 26]. NPs have been proposed to be a nidus where Aβ induces the spread of cortical neurofibrillary degeneration in AD, where mislocalized Tau that has accumulated in dystrophic axons is vulnerable to proteopathic seeds [13]. NPs are characterized by impairment of microtubules, axonal transport and proteostasis [23, 29]. We speculate that aducanumab, through removal of Aβ plaque, might have the downstream impact to restore axonal transport and proteostasis, which could result in reduction of proteopathic Tau seeding and perhaps degradation of some pTau aggregates.

Importantly, the PRIME LTE Patient progressed to moderate dementia during placebo treatment, prior to treatment with aducanumab. Her MMSE score of 14 reflected a more advanced cognitive impairment before starting aducanumab compared the targeted early prodromal and mild AD patients in the PRIME study (MMSE 24.2 + 3.5) [31], and the early AD patients that showed reduced clinical decline on aducanumab 10 mg/kg in the Phase 3 EMERGE study (MMSE 26.3 + 1.68) [6]. The patient’s moderate cognitive impairment indicated that neocortical neurofibrillary degeneration was already established prior to starting aducanumab [19]. Patients treated with AN-1792 [5, 14, 21, 22] similarly had mild-to-moderate AD prior to immunization and all patients progressed to moderate-to-severe dementia with Braak stage V/VI neurofibrillary degeneration despite evidence of plaque reduction and lower NP-Tau and NTs [5, 14, 22]. A hypothetical explanation for progression from moderate-to-severe dementia despite reductions of Aβ plaque and neocortical pTau neuropathology is irreversible activation of downstream neuropathologic cascades leading to progressive synapse impairment or loss such that addressing the disease with an anti-amyloid approach is not sufficient at this late stage in disease.

## Conclusion

In summary, we have presented the first autopsy neuropathology data from an AD patient that was treated with aducanumab after her cognitive impairment had progressed beyond the targeted early AD stages. The neuropathologic findings: (1) corroborate the reduction of Aβ plaques demonstrated by Amyloid PET biomarker data [31], (2) provide evidence of enhanced microglial engagement of Aβ plaques, and (3) provide evidence suggestive of pTau neuropathology reduction that is consistent with Tau PET and CSF and plasma pTau biomarker data in ENGAGE and EMERGE. We found no evidence of significant comorbid neurodegenerative neuropathology and no neuropathologic evidence of adverse treatment effects. This study underscores the critical value of autopsy neuropathology studies to better understand aducanumab’s MOA in the human brain and impact on AD biomarkers in our patients.

## Supplementary Information

Below is the link to the electronic supplementary material.

Supplementary file1 (PDF 3102 kb)
