# Supplementary material for: Alzheimer disease neuropathology in a patient previously treated with aducanumab
Source: Acta Neuropathol. 2022 May 17;144(1):143–53. doi: 10.1007/s00401-022-02433-4 (PMC9217863; doi:10.1007/s00401-022-02433-4)
Supplement: Supplementary file 1 — Supplementary file1 (PDF 3102 kb) [file 401_2022_2433_MOESM1_ESM.pdf]

ONLINE RESOURCE

Title: Alzheimer disease neuropathology in a patient previously treated with aducanumab.

Author List: Edward D. Plowey[1*], Thierry Bussiere[1], Raj Rajagovindan[1], Jennifer Sebalusky[1], Stefan Hamann[1], Christian von Hehn[1], Carmen Castrillo-Viguera[1], Alfred Sandrock[1], Samantha Budd Haeberlein[1], Christopher H. van Dyck[3], Anita Huttner[2]

[1]Research and Development, Biogen, Cambridge, MA and Departments of [2]Pathology and [3]Psychiatry, Yale University School of Medicine, New Haven, CT.

Table S1 Characteristics of untreated AD control cases

Case #	Brain Bank	Age	Gender	*APOE*	Brain Weight (g)	Braak/ NIA A	Braak/ NIA B	NIA C
A17-247	Yale ADRC	96	F	4/4	810	A2	B3	C2
A19-112	Yale ADRC	76	M	2/4	1210	A3	B3	C3
1999-144	NBB	81	F	4/4	1076	C	VI	N/A
2001-099	NBB	82	F	3/3	885	C	V	N/A
2003-024	NBB	75	F	4/4	1009	C	V	N/A
2010-123	NBB	71	M	3/4	1224	C	VI	N/A
2007-068	NBB	70	F	3/3	985	C	VI	N/A
2012-060	NBB	80	F	4/4	1012	C	VI	N/A
2013-040	NBB	70	F	N/A	894	C	VI	N/A

N/A – information not provided with samples, unavailable

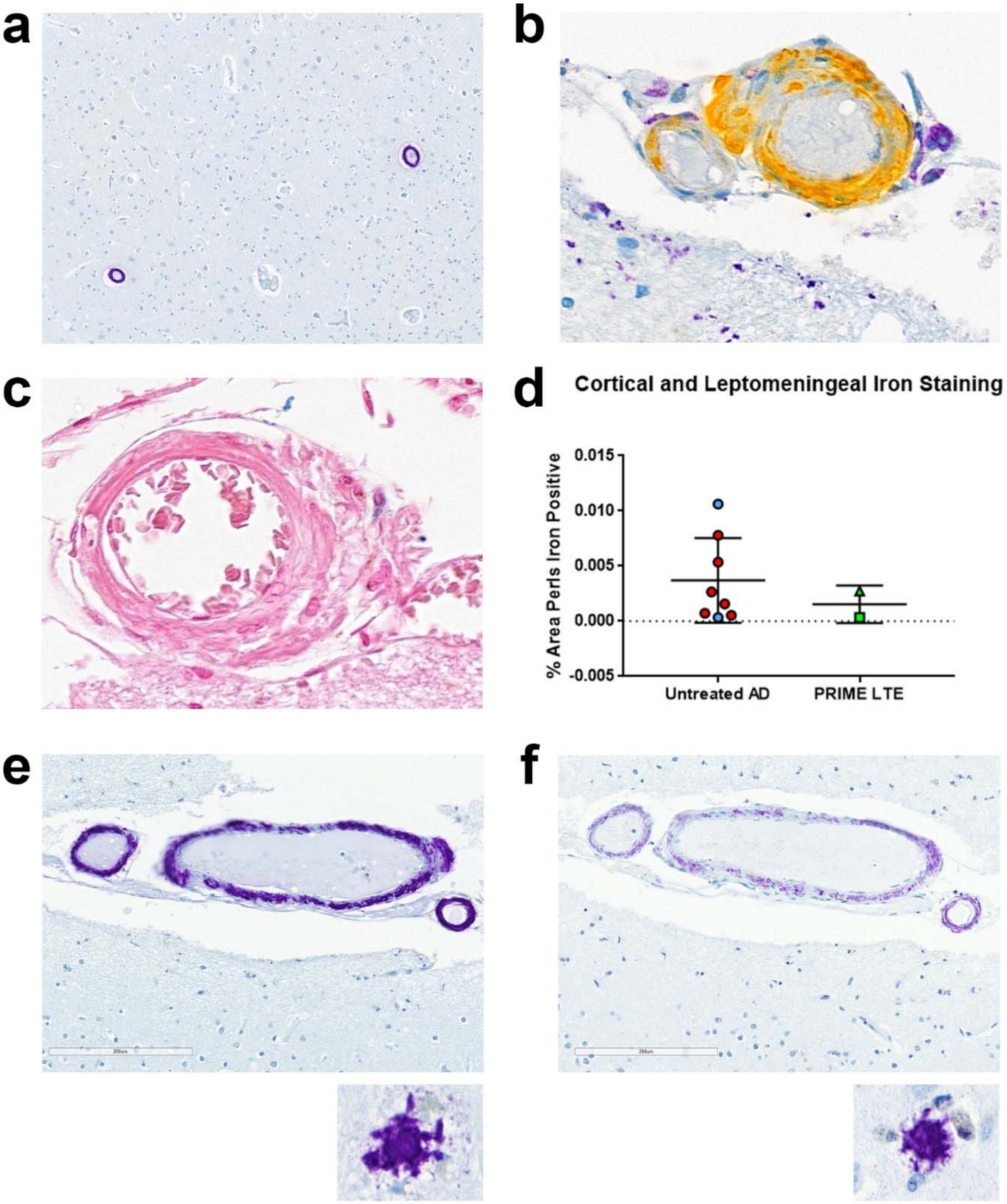

Fig S1 Cerebral amyloid angiopathy (CAA) without evidence of microhemorrhages in the PRIME LTE Patient

a. Low-power image of middle frontal gyrus stained for Aβ (6E10) demonstrating cortical arterioles with CAA otherwise surrounded by cortex devoid of Aβ plaques in the PRIME LTE Patient. **b.** Frontal cortex stained with a duplex IBA1/6E10 immunohistochemical protocol demonstrating a leptomeningeal arteriole with full-thickness mural Aβ deposition but intact mural smooth muscle cells (Vonsattel grade II CAA). There was no significant leptomeningeal/meningovascular inflammation. **c.** Perl's iron stains showed no evidence of superficial siderosis or parenchymal microhemorrhages. **d.** The density of meningovascular and cortical Perl's iron staining was similar in the PRIME LTE subject (square = frontal neocortex; Triangle = temporal neocortex) and a cohort of HIGH AD case controls (blue circles – Yale; red circles – NBB). **e, f.** Vascular Aβ immunoreactivity is higher for 6E10 (**e**) compared to ^{Ch}aducanumab (**f**) whereas similar levels of Aβ immunoreactivity are observed in Aβ plaques.

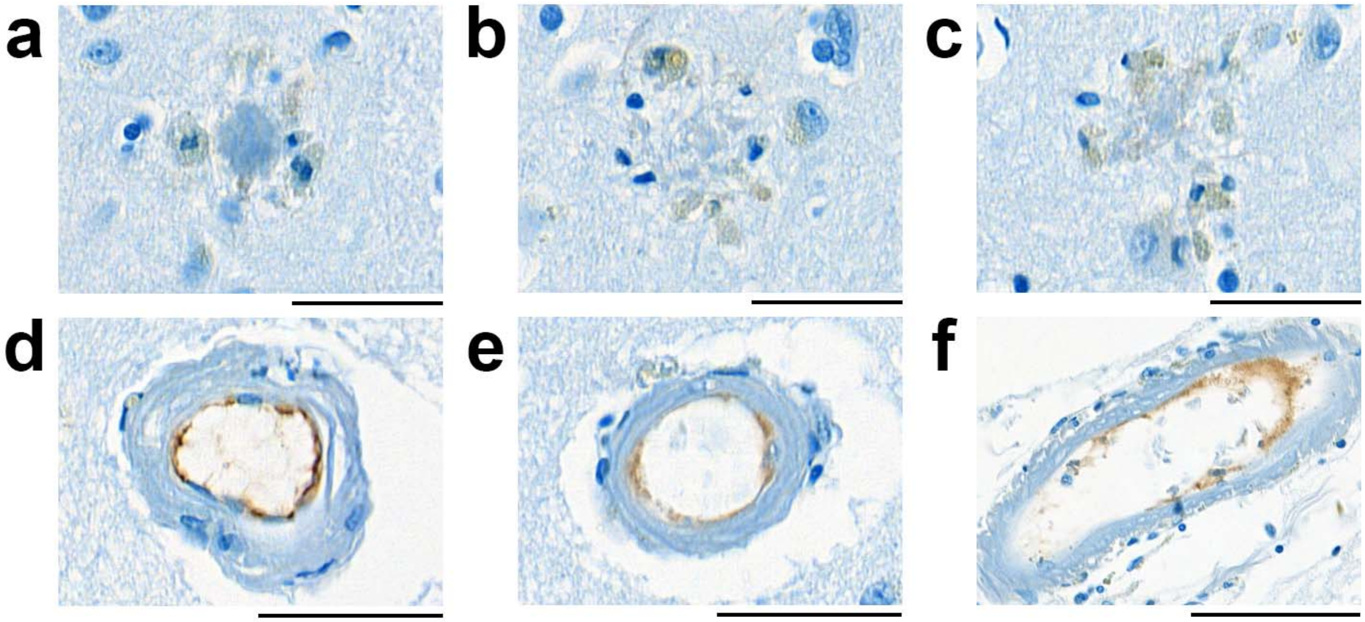

Fig S2 A HuIgG immunohistochemistry assay did not reveal aducanumab in residual plaques in the PRIME LTE Patient

a-c. Representative images of three residual amyloid plaques showed no clear evidence of aducanumab bound to plaque. The assay was first titrated to minimize endogenous parenchymal IgG immunoreactivity in all 9 untreated AD control cases. It is possible that the assay may not have been sensitive enough to detect expected low levels of aducanumab target engagement considering the 4-month interval since the Patient's final aducanumab infusion. Original magnifications 40x, scale bars 40 μm. **d-f.** Representative images of cortical arterioles (d, e) and a leptomeningeal artery (f) showed intravascular IgG immunoreactivity but no clear evidence of aducanumab in the mural amyloid. Original magnifications 40x, scale bars 50 μm (d, e); original magnification 20x, scale bar 100 μm (f).

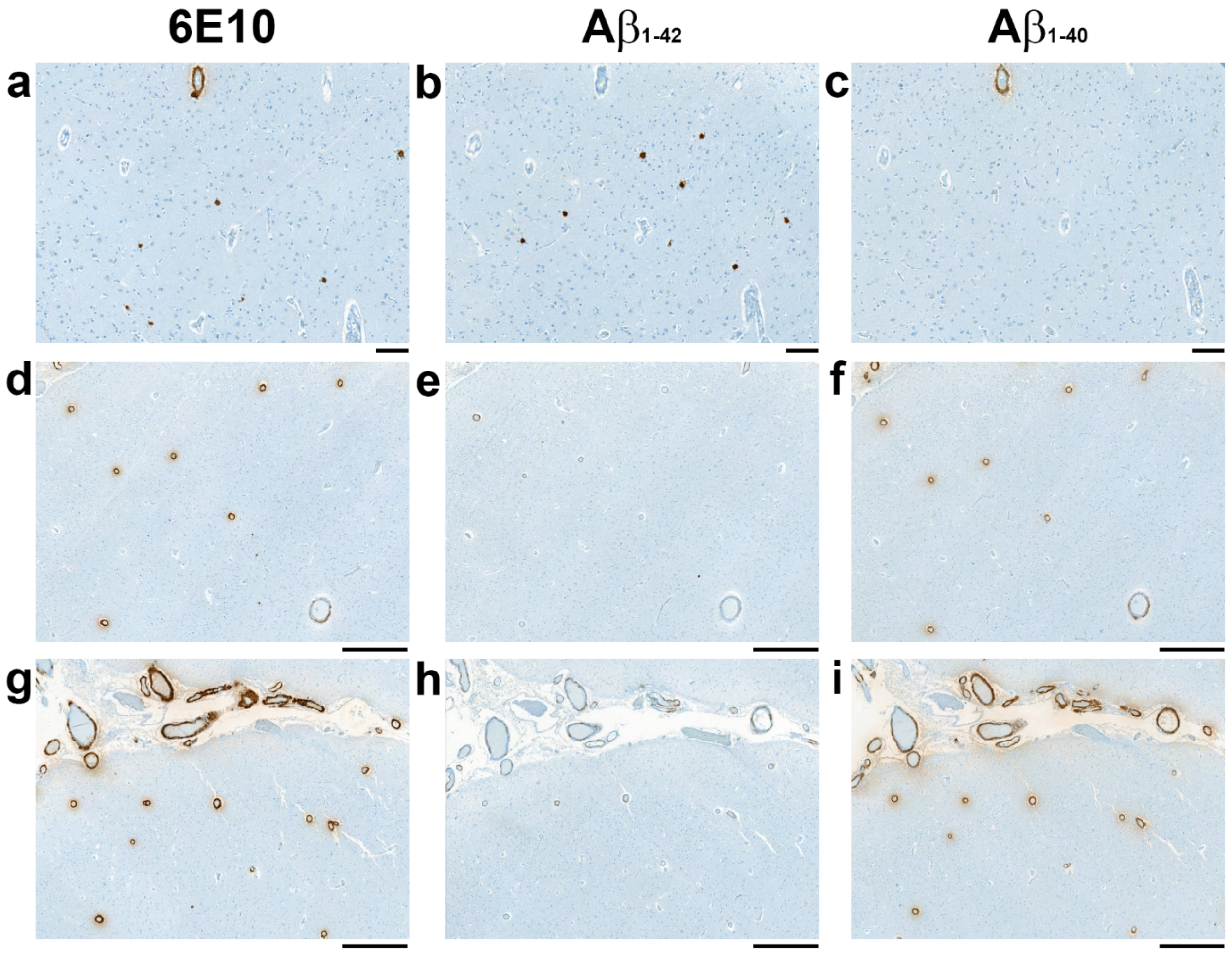

Fig S3 C-terminal Aβ antibody immunoreactivity in the PRIME LTE Patient
a-c. Aβ plaque immunoreactivity/burden was similar between the N-terminal 6E10 antibody protocol (a) and the C-terminal $A\beta_{1\text{-}42}$ antibody protocol (b) but was absent with the $A\beta_{1\text{-}40}$ antibody (c). Original magnification 50x, scale bars 100 μm. **d-f.** Cortical arteriolar Aβ immunoreactivity was similar between the N-terminal 6E10 antibody protocol (d) and the C-terminal $A\beta_{1\text{-}40}$ antibody protocol (f) but was nearly absent with the $A\beta_{1\text{-}42}$ antibody protocol (e). Original magnification 20x, scale bars 100 μm. **g-i.** Leptomeningeal vascular Aβ immunoreactivity was similar between the N-terminal 6E10 antibody protocol (g) and the C-terminal $A\beta_{1\text{-}40}$ antibody protocol (i) but was markedly lower with the $A\beta_{1\text{-}42}$ antibody protocol (h) Original magnification 20x, scale bars 100 μm.

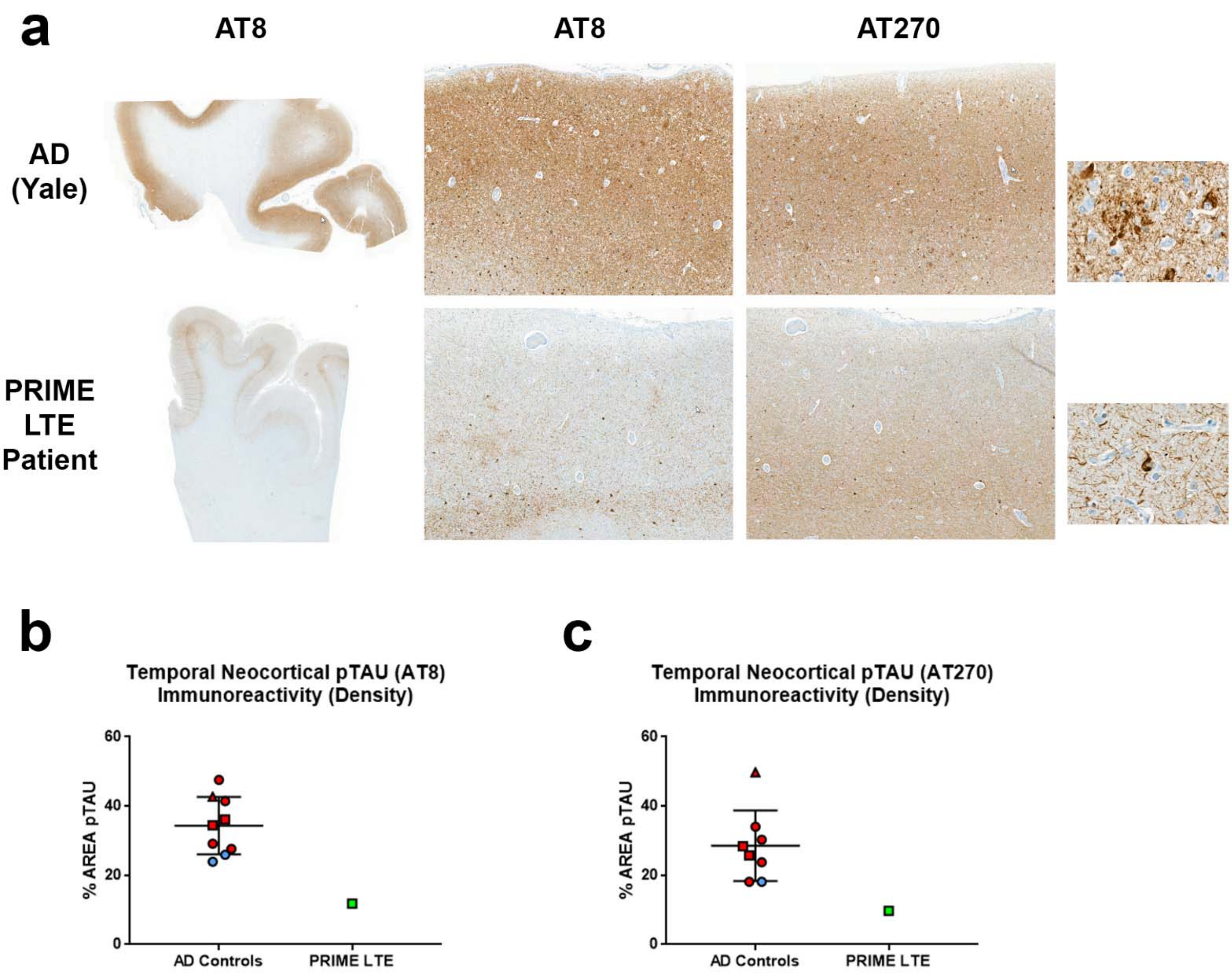

Fig S4 Phosphorylated Tau (AT8 and AT270) immunohistochemistry assays similarly demonstrate lower neurofibrillary pathology in PRIME LTE Patient

a. Sections of frontal neocortex from a HIGH AD neuropathology case control from the Yale ADRC research cohort (top row) and the PRIME LTE Subject (bottom row). Left and middle columns: low power (original magnification 2.5x) and medium power (original magnification 30x) show dense neocortical pTau AT8 immunohistochemical reactivity in the HIGH AD section from Yale compared to the PRIME LTE Subject. Right column: Medium power images demonstrating greater density of neocortical pTau AT270 immunohistochemical reactivity in the HIGH AD section from Yale compared to the PRIME LTE Subject. Inset panels to the right show a neuritic plaque and neurofibrillary tangles in the HIGH AD section from Yale (top). In contrast, AT270 immunoreactive neuritic plaques were not seen in the PRIME LTE Subject (bottom) and the density of neuropil threads was lower, however NFTs were present. **b-c.** Graphical comparisons show low densities of temporal neocortical pTau neuropathology revealed by AT8 IHC (**b**) and AT270 IHC (**c**) in the PRIME LTE Subject compared to a higher range of temporal neocortical pTau neuropathology in a cohort of 9 HIGH AD case controls. Blue datapoints denote samples from Yale; red datapoints indicate samples from NBB. Squares denote *APOE4* non-carriers; circles denote *APOE4* allele carriers; the triangle denotes a sample with unknown *APOE* genotype.
